# The Interaction Between *CRHBP* and *FKBP5* Genes and Childhood Trauma Increases the Risk of Suicide Attempt in Patients with Schizophrenia and Bipolar Disorder

**DOI:** 10.3390/brainsci15111224

**Published:** 2025-11-14

**Authors:** Marco Antonio Sanabrais-Jiménez, Zeltzin Celic Elguea-Ortiz, Ingrid Pamela Morales-Cedillo, Joanna Jiménez-Pavón, Mauricio Rosel-Vales, Ricardo Arturo Saracco-Álvarez, Beatriz Camarena

**Affiliations:** 1 Departamento de Farmacogenética, Subdirección de Investigaciones Clínicas, Instituto Nacional de Psiquiatría Ramón de la Fuente Muñiz, Mexico City 14370, Mexico; masj@inprf.gob.mx (M.A.S.-J.); zeltcelic@gmail.com (Z.C.E.-O.); ingridpmc@gmail.com (I.P.M.-C.); joannajmz@comunidad.unam.mx (J.J.-P.); 2 Dirección de Servicios Clínicos, Instituto Nacional de Psiquiatría Ramón de la Fuente Muñiz, Mexico City 14370, Mexico; m.rosel@inprf.gob.mx; 3 Dirección de Neurociencias, Instituto Nacional de Psiquiatría Ramón de la Fuente Muñiz, Mexico City 14370, Mexico; saracco@inprf.gob.mx

**Keywords:** suicide attempt, *CRHBP*, *FKBP5*, childhood trauma, G × E interaction

## Abstract

**Background/Objectives**: Patients with psychotic disorders have a lifetime risk of suicide attempt (SA) of around 10 to 50%. Genetic variants in the corticotropin-releasing hormone-binding protein (*CRHBP*) and FK506-binding protein 5 (*FKBP5*) genes, which are implicated in the hypothalamic–pituitary–adrenal axis and childhood trauma (CT), are considered risk factors for SA. This study aimed to examine the interaction between the *CRHBP* and *FKBP5* genes and CT in the development of SA. **Methods**: We included 350 patients, 180 patients with schizophrenia and 170 with bipolar disorder. The patients were divided into two groups: 175 with a history of SA and 175 without, and a sample of 350 healthy controls was also included. The Multifactor Dimensionality Reduction program was used to identify G × E interactions between the *CRHBP* (rs7728378, rs10474485, and rs1875999) and *FKBP5* (rs3800373 and rs9296158) and CT in SA. **Results**: The analysis showed that the interaction of *CRHBP* and *FKBP5* with CT increases the risk of presenting at least one SA (OR 4.17; 95% CI [2.67–6.52]; *p* < 0.0001). Additionally, we observed interaction with childhood abuse (OR 4.09; 95% CI [2.61–6.39]; *p* < 0.0001), mainly with emotional (OR 3.67; 95% CI [2.34–5.77]; *p* < 0.0001) and sexual abuse (OR 3.32; 95% CI [2.11–5.23]; *p* < 0.0001). **Conclusions**: Our research indicates that genetic variations in *CRHBP* and *FKBP5* genes and a history of CT increase the probability of presenting at least one SA in patients with schizophrenia and bipolar disorder.

## 1. Introduction

Suicidal behavior is a worldwide public health problem. The World Health Organization estimates more than 720,000 deaths annually due to suicide [1]. Bipolar disorder (BD) and schizophrenia (SCZ) are associated with an increased risk of suicide and suicide attempts (SA) [2,3]. The lifetime prevalence of SA in patients with BD is 34% [4], while it ranges from about 25% to 50% in patients with SCZ [5]. Family, twin, and adoption studies have shown that genetic and environmental factors contribute to suicidal behavior, indicating heritability rates of approximately 30–55% [6,7]. Environmental elements, especially childhood trauma (CT), may influence stress responses in individuals vulnerable to suicidal behavior [8].

As the primary stress-response regulator, the hypothalamic–pituitary–adrenal (HPA) axis coordinates feedforward and feedback inhibition mechanisms among the brain, pituitary, and adrenal glands, thereby regulating cortisol output [9]. Acute stress is marked by increased cortisol levels, which help promote survival as part of the fight-or-flight response [10]. However, prolonged stress exposure reverses these effects, leading to persistent hypercortisolism and a maladaptive stress response, which has been linked to late-onset BD, first-episode psychosis in drug-naïve patients, and suicide behavior [11,12].

The exact neurobiological cause of SA remains unclear; however, numerous studies have demonstrated hyperactivity of the HPA axis [12]. Several genes contribute to this hyperactivation, including those for corticotropin-releasing hormone-binding protein (*CRHBP*) and FK506-binding protein 5 (*FKBP5*). In the pituitary and brain, CRHBP levels are mainly elevated by stress, often in a stressor- and time-dependent way [12].

The corticotropin-releasing hormone (CRH) initiates activation of the hypothalamic–pituitary–adrenal (HPA) axis, while CRH-binding protein (CRHBP) partially regulates its availability by forming a complex with CRH that prevents it from binding to its specific receptors, CRH receptor types 1 and 2 [13]. The increased expression of *CRHBP* appears to act as a mediator of negative feedback by binding CRH and decreasing CRH receptor type 1 activation and signaling [14]; this suggests that changes in *CRHBP* expression could alter the negative feedback that generates hyperactivity in the HPA axis. Another potential mechanism implicated in HPA axis hyperactivation is the decreased gene expression of *FKBP5* [15], which delays the nuclear translocation of the active glucocorticoid receptor complex. Consequently, FKBP5 dampens glucocorticoid signaling and reduces downstream transcriptional responses [16].

The CT has been shown to cause long-lasting changes in the stress response system, which increase the risk of suicidal behavior [17]. CRHBP regulates how much CRH is available outside cells, and lower CRHBP levels have been linked to higher stress reactivity and suicidal behavior in psychiatric patients [13]. FKBP5, which manages glucocorticoid receptor sensitivity, has gene variants that interact with CT to weaken the negative feedback of the HPA axis [18,19]. Although the link between the *FKBP5* gene and trauma has been consistently reported in major depressive disorder and post-traumatic stress disorder, few studies have examined these mechanisms in SCZ and BD or specifically related to SA [20].

The *CRHBP* and *FKBP5* genes play a crucial role in the activation and regulation of the HPA axis and have been associated with SA [21,22]. Nevertheless, the SA represents a complex and heterogeneous behavior shaped by the cumulative effects of multiple low-effect genetic variants, while environmental exposures critically modulate or trigger this genetic vulnerability [23]. The exposure to CT, encompassing different types of neglect and abuse, is one of the primary environmental risk factors associated with the development of SA [13]. Research on gene × environment (G × E) interactions involving *CRHBP*, *FKBP5*, and CT in SA remains limited. Yin et al. [24] analyzed the interaction of *FKBP5*, *SKA2*, *NR3C1*, and CT in SA in European patients with a diagnosis of major depressive disorder, observing interaction between *FKBP5* and CT in the SA. Additionally, Roy et al. [13] explored the interaction between *CRH*, *CRHBP*, *CRHR1*, *CRHR2*, *FKBP5*, and CT in the development of SA in African American patients with a substance use disorder, showing interaction between *CRHBP*, *FKBP5*, and CT. On the other hand, Breen et al. [25] explored the interaction between nineteen genes related to the HPA axis, including *CRHBP*, *FKBP5*, and childhood abuse in SA in patients with BD with Caucasian ancestry, without showing significant results.

Findings concerning the interaction of *CRHBP*, *FKBP5*, and CT in the development of SA are inconclusive; therefore, the present study aimed to analyze the G × E interaction between *CRHBP*, *FKBP5*, and CT in the development of SA in Mexican patients with SCZ and BD.

## 2. Materials and Methods

### 2.1. Sample Selection

This study included 700 Mexican participants, consisting of 350 patients (50%) and 350 healthy controls (50%). The patients were recruited from the Schizophrenia Clinic and Affective Disorders Clinic at the Instituto Nacional de Psiquiatría Ramón de la Fuente Muñiz, meeting DSM-5 criteria for SCZ (n = 180) and for BD type 1 (n = 170). Of the patients, 175 had at least one SA, and 175 did not. In this study, SA was defined as self-destructive behavior intended to end one’s life, regardless of injury severity. Psychiatrists systematically collected information on CT during clinical interviews and documented it in patients’ medical records. A psychiatrist established the operational definition of CT after reviewing all clinical data, classifying them into neglect (emotional and physical) and abuse (emotional, physical, and sexual) according to Bernstein and Fink [26]. No self-report trauma questionnaires (e.g., Childhood Trauma Questionnaire) were used. The inclusion criteria for patients included those with or without at least one SA, with a primary diagnosis of BD or SCZ. Exclusion criteria included patients younger than 18 or those with acute alcohol or substance abuse. Healthy controls were recruited from general hospitals; they had no personal psychiatric history, were over 18, and showed no signs of acute alcohol or substance use. To minimize population stratification, all participants were required to have a Mexican Mestizo ancestry for at least two generations (self-reported ancestry of parents and grandparents).

### 2.2. Ethical Approval

This study adhered to the principles of the Declaration of Helsinki. The protocol received approval from the Ethics Committee of the Instituto Nacional de Psiquiatría Ramón de la Fuente Muñiz (Approval No. CEI/C/015/2019). All participants provided written informed consent prior to enrollment.

### 2.3. Genotyping

Genomic DNA was extracted from peripheral blood samples using the Flexigene DNA kit (Qiagen, Minneapolis, MN, USA). *CRHBP* (rs7728378, rs1875999, and rs10474485) and *FKBP5* (rs3800373 and rs9296158) genetic variants were selected, as they had been previously studied in SA [13,27]. Custom TaqMan assays analyzed genotyping for rs7728378 (C_1399694_20), rs1875999 (C_11433792_10), rs10474485 (C_1399700_20), rs3800373 (C_27489960_10), and rs9296158 (C_1256775_30). Allele-specific genotyping was carried out with TaqMan assays on the ABI Prism^®^ 7500 Sequence Detection System in accordance with the manufacturer’s protocols (Applied Biosystems Inc., Foster City, CA, USA). The final reaction volume was 7 μL and contained 100 ng of genomic DNA, 1× TaqMan Universal Master Mix, and 0.71× SNP Genotyping Assay Mix (Applied Biosystems Inc.). PCR amplification was performed for 40 cycles, following an initial denaturation at 95 °C for 10 min, and then denaturation at 95 °C for 15 s and annealing at 60 °C for 1 min per cycle. All genotyping was conducted in a blinded manner, with respect to the sample information.

### 2.4. Statistical Analyses

Demographics and clinical characteristics were analyzed using the chi-square test, Student’s *t*-test, and ANOVA with Tukey’s multiple comparison post hoc test. All statistical analyses were performed using the program RStudio version 4.3.1 [28].

The power sample calculation analysis was carried out using the R package “gap” version 1.2.3-6 [29], resulting in an estimated power of 0.99 under an additive genetic model, assuming a risk allele frequency of 0.13, an adult Mexican SA prevalence of 3.5% [30], an alpha level of 0.05, and a proportion of patients with SA of 0.5 in a total sample of 350 patients.

Genotype and allele frequencies in controls and patients with and without SA were compared using the chi-square test in Epidat version 3.1. [31], applying a Bonferroni correction for multiple comparisons (five polymorphisms, corrected at *p* < 0.01). Linkage disequilibrium (LD) was estimated based on the patients’ D’ parameter using Haploview version 4.2 [32]. The haplotype effects were assessed using THESIAS(version 3.1.1), with results expressed as haplotypic odds ratios (OR) relative to the most common haplotype [33].

The G × E interactions were analyzed using Multifactor Dimensionality Reduction (MDR) software version 3.0.2 [34] and its permutation testing program version 1.0 beta [35]. MDR is a nonparametric approach for case–control studies of limited size, which detects higher-order nonlinear or non-additive interactions by collapsing multilocus and environmental data into a binary variable: high and low risk [35,36]. Models were evaluated by testing balanced accuracy (TBA), which indicates the correct classification of case/control status, and by cross-validation consistency (CVC), which reflects the stability of the classification. The best interaction models were selected based on TBA values between 0.55 and 0.69, which indicate a meaningful non-additive interaction while avoiding overfitting, together with CVC = 10, which reflects model stability across cross-validation partitions. Statistical significance was confirmed through 1000 permutation tests [34,37].

## 3. Results

### 3.1. Analysis of Demographic and Clinical Characteristics

The analysis of demographic characteristics revealed significant age differences among the group with SA, the group without SA, and the healthy control group (Table 1). The post hoc test showed notable differences between SA patients and healthy controls (*p* < 0.0001), as well as between patients without SA and healthy controls (*p* < 0.0001). Patients with SA were more likely to have comorbid major depressive disorder compared to those without SA (Table 1). Additionally, patients with SA experienced a higher rate of emotional and sexual abuse than patients without SA (Table 1).

### 3.2. Association Analysis

Four of the five genetic variants were in Hardy–Weinberg equilibrium (*p* ≥ 0.05). Moreover, since rs10474485 was not in equilibrium (*p* = 0.004), it was excluded from the analyses. Genotype and allele frequency distributions of *CRHBP* (rs7728378 and rs1875999) and *FKBP5* (rs3800373 and rs9296158) genes are shown in Table 2. There were no differences in *CRHBP* or *FKBP5* gene polymorphisms between SA patients compared with those without SA, SA patients compared with controls, or those without SA patients compared with controls (Table 2).

### 3.3. Haplotype Analysis

**Figure 1:**
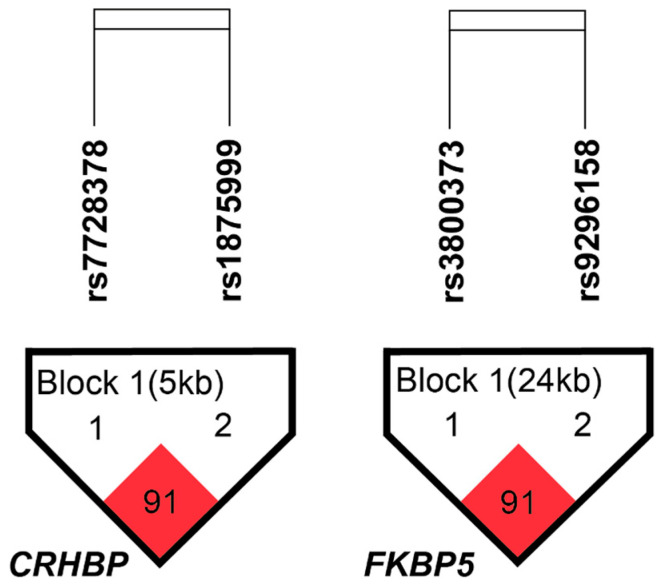
*CRHBP* and *FKBP5* linkage disequilibrium structure. The numbers in the squares refer to pairwise D’ values, while color intensity reflects pairwise r^2^, with darker shading indicating stronger LD. Haploview analysis revealed one LD block in *CRHBP* and one in *FKBP5*, using a within-block average D’ threshold of 0.80. The direction of gene transcription is from left to right.

The *CRHBP* and *FKBP5* haplotype structures are shown in Figure 1. *CRHBP* showed a block composed of rs7728378 and rs1875999 (D’ = 0.91, r^2^ = 0.69), and *FKBP5* showed a block composed of rs3800373 and rs9296158 (D’ = 0.91, r^2^ = 0.71). The haplotype analysis did not reveal any statistically significant differences between subjects with SA and those without SA.

### 3.4. Gene x Gene Interaction Analysis

We analyzed the gene × gene (G × G) interaction for the risk of developing SA between the four loci of the *CRHBP* and *FKBP5* genes (Table 3). We did not observe interaction between *CRHBP* and *FKBP5* gene polymorphisms in the risk of presenting at least one SA (Table 3).

### 3.5. Gene × Environment Interaction Analysis

Table 3 shows the results of G × E interactions between the genetic variants of *CRHBP*, *FKBP5*, and CT in the development of SA. In analyzing *CRHBP* and *FKBP5* gene variants and CT, we found a significant relationship between SA risk (TBA = 0.5714) and an OR of 4.17 (95% CI 2.67–6.52). Additionally, we observed interactions with abuse (TBA = 0.5857; OR 4.09; 95% CI 2.61–6.39), mainly with emotional (TBA = 0.6; OR 3.67; 95% CI 2.34–5.77) and sexual abuse (TBA = 0.5686; OR 3.32; 95% CI 2.11–5.23) concerning the risk of developing SA. All G × E interaction models showed CVC of 10 out of 10 with *p* < 0.0001 (Table 3). Furthermore, exploratory analyses considered sex and primary diagnosis; stratified analyses revealed interactions of *CRHBP* and *FKBP5* gene variants and CT and its subtypes in males, as well as in patients with SCZ, mainly with emotional and sexual abuse (see Supplementary Materials).

**Figure 2:**
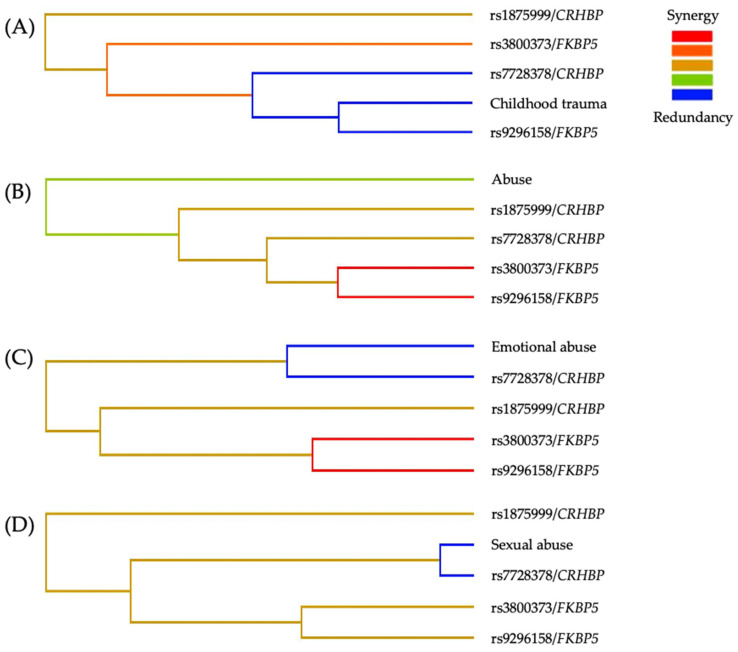
MDR entropy dendrograms of the best interaction models for the risk of SA. (**A**) Childhood trauma, (**B**) Abuse, (**C**) Emotional abuse, and (**D**) sexual abuse. The color gradient represents the direction and magnitude of interaction among variables: red/orange indicates synergistic interactions, green indicates weak or minimal interaction, and blue indicates redundancy. Across models, synergistic clustering between *CRHBP* and *FKBP5* variants is evident, with variation in the specific SNPs involved. CT variables contribute secondary modulatory effects, while sexual abuse shows partial redundancy with rs7728378/*CRHBP*, suggesting convergence of environmental and genetic vulnerability pathways.

Finally, the MDR entropy dendrograms (Figure 2) showed a consistent synergistic interaction between *CRHBP* and *FKBP5* genetic variants across all models associated with SA risk. The CT, abuse, and emotional abuse contributed additional non-additive effects (Figure 2A–C). In contrast, in the sexual abuse model (Figure 2D), a redundancy pattern was observed between sexual abuse and rs7728378/*CRHBP*, while the other redundancies occurred among SNPs within the same genes.

## 4. Discussion

In this study, we evaluated the influence of two genes implicated in the activation and regulation of the HPA axis, along with a history of CT, on the risk of at least one SA among Mexican patients diagnosed with SCZ and BD.

Consistent with previous research included in the meta-analysis by Álvarez et al. [38], the present study shows that major depressive disorder is more frequently seen as a comorbid condition among patients with SCZ and BD who have a history of SA. Similarly, the rates of CT observed in our patients with SA align with those documented in earlier research that used clinical chart reviews to assess this variable in patients with SA [25,39,40]. Our study used a healthy control group from which information related to CT was not obtained. Assessing CT in healthy controls would establish a baseline prevalence and clarify exposure-related risk differences between groups [41], thereby improving the validity and interpretability of the observed associations between CT and SA.

No significant associations were detected between *CRHBP* and *FKBP5* gene polymorphisms and SA. Therefore, our study is consistent with previous reports [13,21,24,42]. Additionally, the haplotype analysis of *CRHBP* and *FKBP5* supports previous negative findings in SA [13,22,42].

Environmental factors, including stressful life events like CT, significantly contribute to triggering or worsening suicidal behavior; however, genetic factors may influence the stress response and, in turn, alter suicide risk [21].

We found a G × E interaction between the *CRHBP* and *FKBP5* genes and CT in SA. Although the evidence is limited, some studies have examined the interaction between candidate genes investigated in our study and CT in the development of SA. In a study of patients with major depressive disorder of European ancestry, the interaction of *FKBP5*, *SKA2*, and *NR3C1* genes with CT in SA was analyzed, showing a significant interaction between *FKBP5* and CT [24]. In a study of African American patients with substance use disorders, the interaction of *CRH*, *CRHBP*, *CRHR1*, *CRHR2*, and *FKBP5* with CT in the development of SA was investigated, identifying significant interaction involving *CRHBP*, *FKBP5*, and CT [13]. However, a sample of Caucasian patients with BD did not show interaction between nineteen genes implicated in the HPA, including *CRHBP* and *FKBP5*, and CT in the development of SA [25]. Latino individuals constitute a genetically heterogeneous population characterized by variable proportions of European, Native American, and African ancestry [43]; therefore, highlighting the need to examine whether the G × E interaction identified in other populations is also present in the Mexican population. Other phenotypic traits that may affect reported findings include differences in primary psychiatric diagnoses, CT criteria, sex, and sample size.

Furthermore, our study examined the G × E interaction between *CRHBP* and *FKBP5*, as well as CT subtypes in SA, demonstrating an interaction involving both candidate genes and childhood abuse, mainly emotional and sexual abuse. Our findings may suggest that *CRHBP* and *FKBP5* genes, along with a subtype of childhood abuse, interact in influencing susceptibility to at least one SA in patients with SCZ and BD. To our knowledge, the interaction between *CRHBP* and *FKBP5*, and emotional or sexual abuse, has not been previously investigated in SA. Therefore, replicating our findings in an independent sample is crucial.

Several genetic variants can influence transcription factor (TF) binding, either by creating a new protein-binding motif or by changing the binding affinity of TFs. These variants may also be located within or near protein-binding motifs, affecting TF-DNA interactions. Therefore, we investigated whether the variants in the candidate genes could impact gene regulation through alterations in TF binding sites (TFBS) by searching JASPAR [44]. For the *CRHBP* gene, the rs7728278 variant was situated within the Arid5a TFBS, while rs1875999 was found within the TFBS for Lef1, Hnf1A, TCF7, and TCF7L2. Regarding the *FKBP5* gene, the rs3800373 SNP was near the FOXD3 and ONECUT1 TFBS (two and seven nucleotides away, respectively), while rs9296158 was within the TFBS for Pou5f::Sox2, ONECUT3, FOXC1, FOXB1, FOXC2, ONECUT1, and GATA1::TAL1. Evidence from both animal models and human studies shows that TFs in the TFBS of *CRHBP* and *FKBP5* are functionally important for neurobiology. Lef1 and TCF family members, like TCF7/TCF7L2, work within the Wnt/β-catenin pathway to control neurogenesis and cortical development [45,46,47,48]. FOXD3 is essential for neural crest formation [49], while Sox2 maintains neural stem cell identity and differentiation [50,51]. Other FOX family members, including FOXB1 and FOXC1, play roles in interneuron differentiation and cerebellar development [52,53], and ONECUT factors (ONECUT1/2/3) are involved in neuronal subtype specification [54,55]. We propose that the genetic variants in TFBS of *CRHBP* and *FKBP5* could alter transcriptional regulation, potentially disrupting HPA axis function. Since the TFs involved are key to neurogenesis and stress-related pathways, such changes might increase vulnerability to stress and SA. These findings support the idea that TFBS-disrupting variants are functional mechanisms [56] that could help us understand the genetic risk for SA.

Regarding the entropy dendrograms, we observed a consistent synergistic interaction between *CRHBP* and *FKBP5* variants across all models. In contrast, redundancies among SNPs within each gene were consistent with expected LD and are therefore not interpreted as independent effects [57]. In contrast, in the model including sexual abuse, a functional redundancy emerged between this environmental variable and rs7728378/*CRHBP*, suggesting a convergence of genetic susceptibility and CT exposure on shared stress-response pathways.

Additionally, from a neurobiological perspective on stress, the interaction between *CRHBP*, *FKBP5*, and CT may contribute to SA risk by causing dysregulation of the HPA axis and glucocorticoid signaling. CRHBP influences extracellular CRH levels and CRHR1 activation [14]; a post-mortem study shows reduced amygdala CRHBP in individuals with BD and SCZ [58], which aligns with increased stress reactivity [59]. FKBP5 lowers glucocorticoid receptor sensitivity, weakening negative feedback and leading to prolonged stress responses [19]. In the presence of CT, these molecular changes may heighten the sensitivity of corticolimbic stress-response circuits, boosting emotional reactivity and diminishing prefrontal regulation [17,20]. Consequently, this neurobiological setup closely associates with impulsivity and quick, threat-driven actions, raising the risk of suicide behavior during intense distress [20,60].

There were certain limitations to our study. First, because ancestry-informative genetic markers were not included, residual population stratification cannot be entirely ruled out, even though the sample was restricted to Mexican Mestizo individuals for at least two generations [61]. Second, the CT information was not collected in the control group. The absence of CT data in the healthy control limits our ability to establish a baseline prevalence of trauma in the general population. However, CT and its subtypes were assessed consistently within the patient sample, reducing the likelihood of differential misclassification between patients with and without SA. Nonetheless, the lack of comparable data in controls limits the evaluation of trauma-related risk gradients beyond the clinical population. Third, the CT was assessed retrospectively from medical records rather than through a standardized psychometric instrument, such as the Childhood Trauma Questionnaire. Although such instruments are highly reliable for evaluating childhood abuse and neglect, previous studies have also validated the use of retrospective clinical reports for this purpose [62,63]. Fourth, our study examined only two genes involved in the HPA axis regulation; nonetheless, SA is a multifactorial behavior influenced by diverse systems, including serotonergic, dopaminergic, noradrenergic, neuroinflammatory, kynurenine, polyamine, lipid metabolism, and endocannabinoid pathways [64]. Fifth, personality traits such as anxiety, aggressiveness, and impulsivity were not assessed; including these dimensions in future studies may help clarify their contribution to SA.

## 5. Conclusions

In summary, patients with SCZ and BD who have a history of CT and carry risk variants in the *CRHBP* and *FKBP5* genes show an increased likelihood of exhibiting at least one SA. Our findings support the hypothesis that alterations in the HPA axis, caused by specific genetic susceptibilities and triggered by stressors such as CT, play a significant role in the development of suicidal behavior. However, these results should be further validated in larger samples of psychiatric patients with SA and extended to include additional genes involved in stress regulation to better understand the contribution of the HPA axis and CT to the etiology of SA.

## Figures and Tables

**Table 1:** Clinic and demographic characteristics of the sample.

Characteristics	Controls (n = 350)	SA (n = 175)	Without SA (n = 175)	Statistics
Age, mean (SD)	55.05 (10.9)	42.71 (13.1)	45.09 (14.65)	F = 71.19, *p* < 0.0001
Sex, n (%)				
Female	207 (69)	110 (62.8)	114 (65.1)	ns
Male	143 (31)	65 (37.1)	61 (34.8)	
Principal diagnosis, n (%)				
Bipolar disorder	-	85 (48.57)	85 (48.57)	ns
Schizophrenia	-	90 (51.43)	90 (51.43)	
Comorbidities, n (%)				
Major depressive disorder	-	19 (10.8)	5 (2.8)	χ^2^ = 7.56, *p* = 0.005
Obsessive–compulsive disorder	-	17 (9.7)	7 (4)	ns
Substance use disorder	-	16 (9.1)	12 (6.8)	ns
Borderline personality disorder	-	6 (3.4)	3 (1.7)	ns
Generalized anxiety disorder	-	5 (2.8)	3 (1.7)	ns
Family history of suicide, n (%)	-	20 (11.4)	14 (8)	ns
Suicide attempt, n (%)				
1	-	102 (58.2)	-	
2	-	33 (18.8)	-	
3	-	20 (11.4)	-	
≥4	-	20 (11.4)	-	
Childhood trauma, n (%)	-	82 (46.8)	44 (25.1)	χ^2^ = 16.97, *p* < 0.0000
Neglect	-	30 (17.1)	21 (12)	ns
Emotional neglect	-	25 (14.2)	20 (11.4)	ns
Physical neglect	-	13 (7.4)	9 (5.1)	ns
Abuse	-	73 (41.7)	39 (22.2)	χ^2^ = 14.29, *p* = 0.0001
Emotional abuse	-	46 (26.2)	26 (14.8)	χ^2^ = 6.31, *p* = 0.01
Physical abuse	-	24 (13.7)	19 (10.8)	ns
Sexual abuse	-	41 (23.4)	15 (8.5)	χ^2^ = 13.28, *p* = 0.0002

ns = no significant.

**Table 2:** Genotype and allele frequencies of *CRHBP* and *FKBP5* variants in patients with and without suicide attempt and controls.

Polymorphism	Genotype			χ^2^, *p*	Allele		χ^2^, *p*
*CRHBP*							
rs7728378	TT	TC	CC		T	C	
SA	78 (0.45)	81 (0.46)	16 (0.09)	2.28, 0.31 ^a^	237 (0.68)	113 (0.32)	0.23, 0.63 ^a^
Without SA	80 (0.46)	71 (0.4)	24 (0.14)	0.56, 0.75 ^b^	231 (0.66)	119 (0.34)	0.5, 0.47 ^b^
Controls	168 (0.48)	153 (0.44)	29 (0.8)	3.8, 0.14 ^c^	489 (0.7)	211 (0.3)	1.61, 0.2 ^c^
rs1875999	CC	CT	TT		C	T	
SA	14 (0.08)	72 (0.41)	89 (0.51)	0.56, 0.75 ^a^	100 (0.29)	250 (0.71)	0.17, 0.67 ^a^
Without SA	18 (0.1)	69 (0.4)	88 (0.5)	0.25, 0.87 ^b^	105 (0.3)	245 (0.7)	0.19, 0.66 ^b^
Controls	24 (0.07)	143 (0.41)	183 (0.52)	1.86, 0.39 ^c^	191 (0.27)	509 (0.73)	0.84, 0.35 ^c^
*FKBP5*							
rs9296158	AA	AG	GG		A	G	
SA	26 (0.15)	81 (0.46)	68 (0.39)	2.2, 0.33 ^a^	133 (0.38)	217 (0.62)	0.74, 0.38 ^a^
Without SA	17 (0.1)	88 (0.5)	70 (0.4)	1.23, 0.53 ^b^	122 (0.35)	228 (0.65)	1.09, 0.29 ^b^
Controls	41 (0.12)	161 (0.46)	148 (0.42)	1.02, 0.6 ^c^	243 (0.35)	457 (0.65)	0.002, 0.96 ^c^
rs3800373	AA	AC	CC		A	C	
SA	77 (0.44)	78 (0.45)	20 (0.11)	0.46, 0.79 ^a^	232 (0.66)	118 (0.34)	0.23, 0.62 ^a^
Without SA	83 (0.48)	72 (0.41)	20 (0.11)	1.84, 0.39 ^b^	238 (0.68)	112 (0.32)	1.38, 0.23 ^b^
Controls	167 (0.48)	155 (0.44)	28 (0.08)	1.76, 0.41 ^c^	489 (0.7)	211 (0.3)	0.37, 0.53 ^c^

^a^ SA compared with without SA. ^b^ SA compared with controls. ^c^ Without SA compared with controls.

**Table 3:** Best models for the risk of suicide attempt in patients with schizophrenia and bipolar disorder between *CRHBP*, *FKBP5*, and childhood trauma.

Interaction Models	TBA	OR (95% CI)	CVC	*p*
Gene × Gene				
*CRHBP* and *FKBP5*	0.5171	2.2 (1.41–3.43)	10/10	0.0004
Gene × Environment				
*CRHBP*, *FKBP5*, and childhood trauma ^a^	0.5714	4.17 (2.67–6.52)	10/10	<0.0001
*CRHBP*, *FKBP5*, and neglect	0.52	2.54 (1.65–3.92)	10/10	<0.0001
*CRHBP*, *FKBP5*, and emotional neglect	0.5171	2.41 (1.57–3.71)	10/10	<0.0001
*CRHBP*, *FKBP5*, and physical neglect	0.4886	2.48 (1.6–3.84)	10/10	<0.0001
*CRHBP*, *FKBP5*, and abuse ^a^	0.5857	4.09 (2.61–6.39)	10/10	<0.0001
*CRHBP*, *FKBP5*, and emotional abuse ^a^	0.6	3.67 (2.34–5.77)	10/10	<0.0001
*CRHBP*, *FKBP5*, and physical abuse	0.5229	2.87 (1.85–4.43)	10/10	<0.0001
*CRHBP*, *FKBP5*, and sexual abuse ^a^	0.5686	3.32 (2.11–5.23)	10/10	<0.0001

*CRHBP* (rs7728378 and rs1875999), *FKBP5* (rs3800373 and rs9296158). TBA = Testing balanced accuracy; OR = Odds ratio; CI = Confidence interval; CVC = Cross-validation consistency; 1000-fold permutation test. ^a^ Best models of interaction in SA.

## Data Availability

The data presented in this study are available on request from the corresponding author due to restrictions in data availability, as the dataset is not deposited in a public repository.

## Supplementary Materials

The following supporting information can be downloaded at: https://www.mdpi.com/article/10.3390/brainsci15111224/s1, Table S1. Best models for the risk of suicide attempt in female patients with schizophrenia and bipolar disorder between *CRHBP*, *FKBP5*, and childhood trauma. Table S2. Best models for the risk of suicide attempt in male patients with schizophrenia and bipolar disorder between *CRHBP*, *FKBP5*, and childhood trauma. Table S3. Best models for the risk of suicide attempt in patients with schizophrenia between *CRHBP*, *FKBP5*, and childhood trauma. Table S4. Best models for the risk of suicide attempt in patients with bipolar disorder between *CRHBP*, *FKBP5*, and childhood trauma.

## Abbreviations

The following abbreviations are used in this manuscript:

BD	Bipolar disorder
CI	Confidence interval
CRH	Corticotropin-releasing hormone
CRHBP	CRH-binding protein
CT	Childhood trauma
CVC	Cross-validation consistency
FKBP5	FK506-binding protein 5
G × E	Gene × environment
G × G	Gene × gene
HPA	Hypothalamic–pituitary–adrenal
MDR	Multifactor dimensionality reduction
OR	Odds ratio
SA	Suicide attempt
SCZ	Schizophrenia
TBA	Testing balanced accuracy
TF	Transcription factor
TFBS	TF binding sites
