# Supplementary material for: The Interaction Between CRHBP and FKBP5 Genes and Childhood Trauma Increases the Risk of Suicide Attempt in Patients with Schizophrenia and Bipolar Disorder
Source: Brain Sci. 2025 Nov 14;15(11):1224. doi: 10.3390/brainsci15111224 (PMC12650206; doi:10.3390/brainsci15111224)
Supplement: Supplementary file 1 [file brainsci-15-01224-s001.zip › brainsci-3964180-supplementary.pdf]

Table S1. Best models for the risk of suicide attempt in female patients with schizophrenia and bipolar disorder between *CRHBP*, *FKBP5*, and childhood trauma.

Interaction models	TBA	OR (95% CI)	CVC	p
Gene x Gene				
CRHBP and *FKBP5*	0.4787	2.45 (1.39 – 4.32)	10/10	0.0016
Gene x Environment				
CRHBP, *FKBP5*, and childhood trauma	0.4699	3.9 (2.23 – 6.84)	10/10	< 0.0001
CRHBP, *FKBP5*, and neglect	0.4746	2.82 (1.63 – 4.87)	10/10	0.0002
CRHBP, *FKBP5*, and emotional neglect	0.4834	2.84 (1.64 – 4.9)	10/10	0.0001
CRHBP, *FKBP5*, and physical neglect	0.4888	2.82 (1.6 – 4.97)	10/10	0.0003
CRHBP, *FKBP5*, and abuse	0.4872	3.86 (2.2 – 6.77)	10/10	< 0.0001
CRHBP, *FKBP5*, and emotional abuse	0.4782	3.57 (2.03 – 6.27)	10/10	< 0.0001
CRHBP, *FKBP5*, and physical abuse	0.5053	3.33 (1.9 – 5.82)	10/10	< 0.0001
CRHBP, *FKBP5*, and sexual abuse	0.4909	3.36 (1.94 – 5.83)	10/10	< 0.0001

CRHBP (rs7728378 and rs1875999), *FKBP5* (rs3800373 and rs9296158). TBA = Testing balanced accuracy; OR = Odds ratio; CI = Confidence interval; CVC = Cross-validation consistency; 1000-fold permutation test. [a]Best models of interaction in SA.

Table S2. Best models for the risk of suicide attempt in male patients with schizophrenia and bipolar disorder between *CRHBP*, *FKBP5*, and childhood trauma.

Interaction models	TBA	OR (95% CI)	CVC	p
Gene x Gene				
CRHBP and *FKBP5*[a]	0.5874	5.05 (2.36 – 10.81)	10/10	< 0.0001
Gene x Environment				
CRHBP, *FKBP5*, and childhood trauma[a]	0.5869	11.52 (4.98 – 26.68)	10/10	< 0.0001
CRHBP, *FKBP5*, and neglect[a]	0.5936	6.23 (2.87 – 13.51)	10/10	< 0.0001
CRHBP, *FKBP5*, and emotional neglect[a]	0.5859	5.78 (2.68 – 12.45)	10/10	< 0.0001
CRHBP, *FKBP5*, and physical neglect[a]	0.6177	6.32 (2.91 – 13.75)	10/10	< 0.0001
CRHBP, *FKBP5*, and abuse[a]	0.6033	10.28 (4.52 – 23.37)	10/10	< 0.0001
CRHBP, *FKBP5*, and emotional abuse[a]	0.5849	7.94 (3.58 – 17.57)	10/10	< 0.0001
CRHBP, *FKBP5*, and physical abuse[a]	0.6156	7.34 (3.34 – 16.14)	10/10	< 0.0001
CRHBP, *FKBP5*, and sexual abuse[a]	0.6284	7.45 (3.31 – 16.77)	10/10	< 0.0001

CRHBP (rs7728378 and rs1875999), *FKBP5* (rs3800373 and rs9296158). TBA = Testing balanced accuracy; OR = Odds ratio; CI = Confidence interval; CVC = Cross-validation consistency; 1000-fold permutation test. [a]Best models of interaction in SA.

Table S3. Best models for the risk of suicide attempt in patients with schizophrenia between *CRHBP*, *FKBP5*, and childhood trauma.

**Interaction models**	**TBA**	**OR (95% CI)**	**CVC**	**p**
Gene x Gene				
CRHBP and *FKBP5*	0.5444	4.12 (2.19 – 7.74)	10/10	< 0.0001
Gene x Environment				
CRHBP, *FKBP5*, and childhood trauma[a]	0.5833	8.27 (4.21 – 16.24)	10/10	< 0.0001
CRHBP, *FKBP5*, and neglect	0.4944	5.01 (2.61 – 9.6)	10/10	< 0.0001
CRHBP, *FKBP5*, and emotional neglect	0.5	4.7 (4.47 – 8.96)	10/10	< 0.0001
CRHBP, *FKBP5*, and physical neglect	0.5278	4.57 (2.42 – 8.64)	10/10	< 0.0001
CRHBP, *FKBP5*, and abuse[a]	0.6	7.75 (3.97 – 15.11)	10/10	< 0.0001
CRHBP, *FKBP5*, and emotional abuse[a]	0.5611	6.76 (3.52 – 12.97)	10/10	< 0.0001
CRHBP, *FKBP5*, and physical abuse	0.5278	4.57 (2.42 – 8.64)	10/10	< 0.0001
CRHBP, *FKBP5*, and sexual abuse[a]	0.5889	7.09 (3.5 – 14.38)	10/10	< 0.0001

CRHBP (rs7728378 and rs1875999), *FKBP5* (rs3800373 and rs9296158). TBA = Testing balanced accuracy; OR = Odds ratio; CI = Confidence interval; CVC = Cross-validation consistency; 1000-fold permutation test. [a]Best models of interaction in SA.

Table S4. Best models for the risk of suicide attempt in patients with bipolar disorder between *CRHBP*, *FKBP5*, and childhood trauma.

**Interaction models**	**TBA**	**OR (95% CI)**	**CVC**	**p**
Gene x Gene				
CRHBP and *FKBP5*	0.3765	2.27 (1.17 – 4.4)	10/10	0.01
Gene x Environment				
CRHBP, *FKBP5*, and childhood trauma	0.4941	5.12 (2.63 – 9.95)	10/10	< 0.0001
CRHBP, *FKBP5*, and neglect	0.4294	3.2 (1.71 – 6.01)	10/10	0.0002
CRHBP, *FKBP5*, and emotional neglect	0.4235	3.19 (1.7 – 5.98)	10/10	0.0002
CRHBP, *FKBP5*, and physical neglect	0.4059	2.7 (1.43 – 5.07)	10/10	0.001
CRHBP, *FKBP5*, and abuse	0.4882	4.82 (2.46 – 9.47)	10/10	< 0.0001
CRHBP, *FKBP5*, and emotional abuse	0.4412	3.6 (1.9 – 6.8)	10/10	< 0.0001
CRHBP, *FKBP5*, and physical abuse	0.3647	2.75 (1.48 – 5.14)	10/10	0.001
CRHBP, *FKBP5*, and sexual abuse	0.4235	3.72 (1.81 – 7.62)	10/10	0.0002

CRHBP (rs7728378 and rs1875999), *FKBP5* (rs3800373 and rs9296158). TBA = Testing balanced accuracy; OR = Odds ratio; CI = Confidence interval; CVC = Cross-validation consistency; 1000-fold permutation test. [a]Best models of interaction in SA.
